# Dose‐ and pattern‐ physical activity is associated with lower risk of dementia

**DOI:** 10.1002/alz70860_099227

**Published:** 2025-12-23

**Authors:** Yan Wang, Fangyu Li, Jianping Jia

**Affiliations:** ^1^ Innovation Center for Neurological Disorders and Department of Neurology, Xuanwu Hospital, Capital Medical University, Beijing, Beijing, China; ^2^ Innovation Center for Neurological Disorders, Department of Neurology, Xuan Wu Hospital, Capital Medical University, Beijing, Beijng, China

## Abstract

**Background:**

The amount and patterns of physical activity to benefit cognitive health remains unclear.

**Method:**

In UK Biobank cohort, individuals with a full week of accelerometer‐based moderate‐to‐vigorous PA (MVPA) and light PA (LPA) data were analysis. The last date for dementia was April 2024. Associations between the incidence of all‐cause dementia, Alzheimer's disease (AD), and Vascular dementia (VaD), and the level and patterns of PA were assessed using Cox proportional hazards regression models. These including: 1) MVPA gradients were compared with less than 150 min/week; 2) Compared active intensive (≥50% of MVPA in 1–2 days), active regular (not meeting active intensive), and inactive (<300 min/week); 3) performed stratified analyses by age, sex and *APOE ε*4 carrier status; 4) Investigated the effect of LPA among inactive MVPA.

**Result:**

91512 individuals (mean [SD] age, 56.03[7.8] years; 55.9% female) were included. Compared with <150 min, 150–299 min of moderate to vigorous physical activity (MVPA) in weekend or regular pattern did not have significantly lower dementia incidence, but over 300 min/week had. When stratified at 300 min/week of MVPA, hazard ratios for dementia were 0.73 (95% CI: 0.60–0.89) for weekend pattern and 0.79 (95% CI: 0.64–0.98) for regular pattern. For inactive MVPA, >840 min/week of light physical activity (LPA) was associated with lower dementia incidence.

**Conclusion:**

More than 300 min/week of MVPA, whether concentrated within 1 to 2 days or distributed evenly, was associated with decreased risk of dementia. LPA partially compensated inactive MVPA.

